# The Mirage of Factor Equivalence: Examining the Complexities of Non‐Factor Therapies in Haemophilia

**DOI:** 10.1111/hae.70131

**Published:** 2025-09-16

**Authors:** Yesim Dargaud, Arianna Colombo, Maria Elisa Mancuso

**Affiliations:** ^1^ UR4609 Hemostasis &Thrombosis Faculty of Medicine Lyon East University of Lyon 1 Lyon France; ^2^ French Reference Centre For Hemophilia Louis Pradel Hospital Lyon France; ^3^ Center For Thrombosis and Hemorrhagic Diseases IRCCS Humanitas Research Hospital Rozzano Milan Italy; ^4^ Humanitas University Pieve Emanuele Milan Italy

**Keywords:** equivalence, Factor VIII, mimetics, non‐factor therapy, rebalancing agents

## Abstract

**Background:**

This article provides a critical analysis of the ‘factor equivalence’ concept as applied to non‐factor therapies (NFTs) for haemophilia, highlighting its limitations and clinical implications. Although factor equivalence estimates serve as a comparative tool for evaluating pro‐coagulant effects across different therapies, they may fail to accurately represent the true haemostatic potential or clinical efficacy due to the diverse mechanisms of action of different molecules and patient‐specific factors.

**Aim:**

The article challenges the widespread adoption of factor equivalence as a universal benchmark to quantify the haemostatic potential of NFTs.

**Discussion and Conclusion:**

It advocates for patient‐ and context‐specific laboratory assessments that reflect each therapy's unique pharmacological profile. This approach is particularly relevant to enhance personalised treatment strategies, especially in high‐risk situations such as surgery or severe breakthrough bleeding, thereby optimising the use of novel haemostatic therapies.

## 1 Introduction

The therapeutic landscape of haemophilia has been significantly transformed with the advent of non‐factor therapies (NFTs). Traditionally managed with Factor VIII (FVIII) or Factor IX (FIX) concentrates, treatment choices for prophylaxis have been enriched with novel agents able to prevent the majority of bleeding episodes with a stable and consistent level of protection, reduced treatment burden and improved quality of life [1]. NFTs include subcutaneous activated FVIII (FVIIIa) mimetics, as well as the so‐called rebalancing agents that target natural anticoagulants such as tissue factor pathway inhibitor (TFPI) and antithrombin (AT) [1]. Each molecule has a distinct pharmacological profile, but they all enhance thrombin generation. Emicizumab, the first approved FVIIIa mimetic, is a bispecific monoclonal antibody able to bridge activated FIX (FIXa) and Factor X (FX) to partially mimic FVIIIa activity and enhance thrombin generation [2]; fitusiran is a small‐interference RNA (siRNA) able to prevent AT synthesis [3]; concizumab and marstacimab are monoclonal antibodies able to bind and inhibit TFPI [4, 5]. Clinical and real‐world data show remarkably reduced bleeding rates across different patient populations as compared with episodic treatment, prompting their use to optimise or even ensure regular prophylaxis to many [6, 7, 8, 9, 10]. Despite their clinical benefits, NFTs pose the challenge of laboratory assessment of their clotting activity because, at variance with FVIII and FIX, they are not clotting factors. Therefore, the haemostatic potential conferred by these agents remains difficult to be quantified. Although they all enhance thrombin generation, the precise extent of this effect is not fully defined, and therefore the risk of breakthrough bleeds remains unpredictable at the individual level. This uncertainty arises for several reasons. First, routine laboratory assays commonly used in hospital laboratories are not capable of accurately assessing the haemostatic activity of NFTs. Second, the complex mechanisms of action of these therapies, involving multiple proteins, further complicate assessment. Finally, individual responses to these therapies can vary significantly between patients. To aid clinical interpretation, researchers have attempted to define the concept of FVIII/FIX equivalence for NFT, based on in vitro data and in vivo studies in animal models. This framework has been broadly adopted by clinicians as a reference to guide therapeutic decisions. Yet, it remains uncertain whether the term ‘equivalence’ is truly appropriate in this context or if this is only a mirage we are all longing to.

## 2 The Concept of ‘Factor Equivalence’

Owing to the similar mechanism of action between FVIII and emicizumab (i.e., bridging FIX and FX), the concept of factor equivalence has first emerged as a practical framework to quantify the haemostatic effect of emicizumab and translate it into an estimated equivalent FVIII clotting activity. In fact, clotting factor activity levels have been used for decades as an important benchmark by both clinicians and patients to quantify the level of protection against bleeds in different settings. The assumed protective range of 9%–20% FVIII activity at steady state with emicizumab stems from models rather than objectively measured clinical validation [11, 12]. Similarly, the possibility to estimate FVIII equivalence also for rebalancing agents as marstacimab has been explored in a mouse model [13]. According to the Cambridge Dictionary, ‘equivalence’ refers to ‘the fact of having the same amount, value, purpose, qualities, etc.’. This definition implies a degree of sameness or interchangeability that may not fully apply to the comparison between NFT and clotting factors.

### 2.1 Limitations of Equivalence: Biological Differences Between FVIII and Emicizumab

The fundamental biochemical differences between emicizumab and FVIII have been thoroughly outlined by Lenting et al. [14]. The authors emphasise that emicizumab and FVIII are distinct proteins governed by different regulatory mechanisms. Key distinguishing features include:

1. Distinct binding topology – FVIIIa engages multiple sites on both FIXa and FX, whereas emicizumab binds single epitopes on the EGF1 domain of FIX(a) and the EGF2 domain of FX(a).
2. Lower binding affinity – Emicizumab binds its targets with micromolar affinity, which is significantly lower than that of FVIIIa.
3. Partial cofactor function – FVIIIa not only bridges FIXa to FX but also stabilises the FIXa active site and enhances phospholipid surface binding; emicizumab solely mediates bridging.
4. Molar ratio and regulation – FVIIIa circulates in plasma at low levels relative to those of FIXa and FX and is tightly regulated via activation/inactivation mechanisms. In contrast, emicizumab is present in molar excess and lacks dynamic on/off regulation.
5. Lack of activation specificity – FVIIIa interacts exclusively with FIXa and non‐activated FX, while emicizumab binds both activated and non‐activated forms of both factors.

In addition, beyond its role in coagulation, FVIII also participates in several extra‐coagulant processes that are not replicated by emicizumab: (i) Studies have implicated FVIII in bone metabolism, potentially through effects on osteoblast function and bone remodelling pathways [15]; (ii) FVIII and von Willebrand factor (VWF) also interact directly with platelets contributing to thrombus formation and stabilisation through mechanisms distinct from its cofactor activity [16, 17]; (iii) FVIII may influence vascular endothelial function, with suggested roles in maintaining endothelial integrity and modulating inflammatory responses. These pleiotropic effects highlight the biological complexity of FVIII and further differentiate it from emicizumab and other NFT. Given these additional functions, the concept of ‘equivalence’ between FVIII and emicizumab becomes increasingly difficult to justify.

### 2.2 Limitations of Equivalence: Haemostatic Differences Between FVIII and Emicizumab

The commonly accepted equivalence of 9%–20% FVIII activity for emicizumab does not fully reflect the clinical efficacy seen in all patients on emicizumab prophylaxis. Patients with mild haemophilia A, with FVIII levels between 9% and 20%, generally show a consistent haemorrhagic phenotype, meaning they do not experience spontaneous bleeding. This is true for most patients, except those with discrepancies between one‐stage and chromogenic FVIII assays, or those with FVIII activity below 10% in one of the assays – typically observed with the chromogenic assay. What is observed in patients receiving emicizumab prophylaxis contrasts with the clinical haemorrhagic profile of haemophilia patients with 9%–20% FVIII activity. It has been reported that some patients receiving emicizumab prophylaxis experience spontaneous muscle haematomas [18], joint bleeds [19] or even intracranial haemorrhages [20]. Although the majority of these patients exhibit thrombin generation similar to that of mild haemophilia [21], a subset shows thrombin generation comparable to the general population. However, those who experience spontaneous bleeding tend to have lower thrombin generation, resembling the levels typically observed in moderate or severe haemophilia patients [20].

The same murine models used to estimate the FVIII equivalence of emicizumab have also been employed to investigate in vivo clot formation induced by recombinant FVIII (rFVIII), rFVIIIFc and emicizumab, with a particular focus on the structure of the fibrin network. The different mode of action of emicizumab alters the kinetics of fibrin and FXIIIa formation, resulting in distinct clot morphologies. Structural differences were observed between clots formed with 10% FVIII and those induced by emicizumab, with emicizumab‐treated mice exhibiting a greater tendency for rebleeding [22]. This is another difference that highlights the contradiction to the FVIII equivalence that clinicians have perceived.

### 2.3 Limitations of Models for Determining Factor VIII Equivalence

Emicizumab FVIII equivalence of 10% was described in an FVIII‐deficient mice model infused with a cocktail containing human FIX (hFIX) and FX (both 100 U/kg), as emicizumab cannot bind to murine FIX and FX. FVIII equivalence was determined in this emicizumab‐responsive semi‐humanised mouse model, using a tail‐clip bleeding model.

Tail vein bleeding in mice is the most commonly used test to assess haemostatic function, but it presents significant challenges, particularly in standardisation. The International Society on Thrombosis and Haemostasis (ISTH) has highlighted subtle variables, such as the injury site, method of infliction and blood collection techniques, that can impact results [23]. Even if standardised, the test may not detect critical bleeding conditions, as temporary clot stability during vascular spasm could be enough to prevent bleeding. These limitations call for caution when drawing conclusions about bleeding diathesis based on a single model or set of conditions.

**FIGURE 1:**
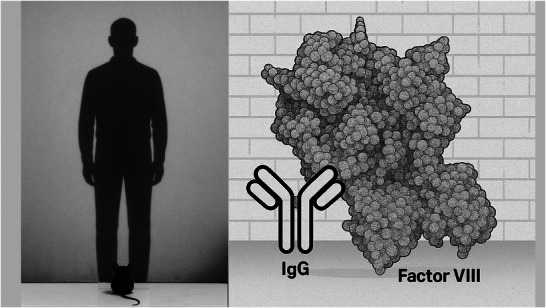
Symbolic illustration of the limitations of murine models in translational haemostasis research. Despite sharing over 90% genomic homology with humans and playing a pivotal role in mechanistic and preclinical research, murine models exhibit inherent limitations in predicting clinical responses to therapeutic agents such as bispecific antibodies bridging Factor IXa to Factor X, or antibodies directed against natural anticoagulants. Although these molecules are designed to mimic coagulation factor activity or modulate the haemostatic balance, they are not coagulation factors per se. Therefore, interpreting them as true functional equivalents based on equivalence observed in animal models may lead to misleading clinical interpretations. IgG and Factor VIII (FVIII) molecules are represented schematically and are not shown to scale.

In addition, translating results from animal models to clinical practice remains a significant challenge. Although murine models have been invaluable in dissecting coagulation mechanisms and assessing the efficacy and safety of novel therapies, their ability to predict human responses is limited by interspecies differences in physiology, immunogenicity and the regulation of coagulation pathways. A notable example is the development of rebalancing agents. Preclinical studies of anti‐TFPI therapies in haemophilic rabbits [24] and AT‐lowering siRNA in haemophilic mice showed promising haemostatic efficacy without thrombotic complications [25], supporting further clinical development. However, early‐phase human trials were impacted by thrombotic events, resulting in the temporary suspension of clinical trial programs. These discrepancies underscore the critical need for cautious interpretation when attempting to extrapolate clinical equivalence from preclinical models (Figure 1).

### 2.4 The Estimated Equivalence in Models Without Clinical Validation Overlooks the Crucial Aspect: The Individual

**FIGURE 2:**
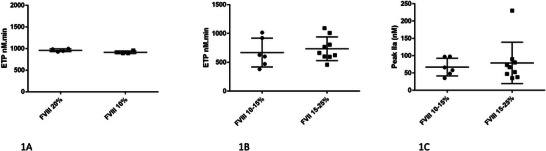
In vitro and ex vivo thrombin generation in plasma from patients with severe haemophilia A. Comparison of different Factor VIII (FVIII) products added to a uniform pool of FVIII‐deficient plasma. Endogenous thrombin potential values measured after in vitro supplementation of pooled plasma samples with 10% or 20% FVIII clotting activity (A); Endogenous thrombin potential (B) and thrombin peak (C) measured ex vivo in patients with 10% or 20% circulating FVIII activity levels. Data were generated by the authors to illustrate that, although all FVIII concentrates induce a similar improvement in thrombin generation in vitro, it is not possible to assume an identical haemostatic effect in vivo in all patients with the same FVIII level due to interindividual variability. The observed marked interindividual variability, despite identical FVIII activities, highlights that FVIII equivalence does not consistently reflect the haemostatic phenotype. Therefore, equating emicizumab to 9%–20% FVIII has limited clinical relevance. Thrombin generation was measured in platelet‐poor plasma using the Calibrated Automated Thrombin Generation Assay (CAT) with a low tissue factor concentration of 1 pM (PPP Low reagent, Stago, Asnières‐sur‐Seine, France).

Referring to an equivalence range of 9%–20% implies a clinically homogeneous profile in terms of haemostatic capacity and bleeding tendency. This is not correct. In fact, interindividual differences in coagulation potential can be observed among patients who receive identical doses of different therapeutic FVIII molecules with equivalent procoagulant efficacy. This is because FVIII is not the sole determinant of overall haemostatic capacity. Variability in the activity levels of other coagulation factors – ranging from 50% to 150% – as well as interindividual differences in platelet procoagulant activity represents important sources of heterogeneity. This variability is exemplified in Figure 2. When comparing the in vitro improvement of thrombin generation in a pool of FVIII‐deficient plasma supplemented with 10% or 20% exogenous FVIII – whether plasma‐derived, recombinant, extended half‐life (EHL) or ultra‐EHL FVIII – the enhancement is nearly identical across all FVIII concentrates in terms of endogenous thrombin potential (area under the thrombin generation curve) (Figure 2A). In such a model, the FVIII‐equivalent activity of emicizumab at the 10%–20% level corresponds to a clearly defined haemostatic profile. When thrombin generation is assessed ex vivo in patients who have received an infusion of FVIII concentrate and exhibit circulating FVIII levels of 10% or 20%, a substantial interindividual variability in coagulation potential can be observed despite identical FVIII levels (Figure 2B, C). This model, which integrates patient‐specific biological characteristics, highlights that FVIII equivalence does not systematically reflect a consistent haemostatic phenotype. Therefore, stating that emicizumab corresponds to 9%–20% FVIII has limited clinical relevance and should be interpreted with caution.

### 2.5 Rebalancing Agents and Their Factor Equivalence

#### 2.5.1 Fitusiran

In FVIII‐deficient mice, fitusiran significantly reduced bleeding in a saphenous vein bleeding model, with efficacy comparable to 25 IU/kg FVIII (∼50% activity), although no FVIII equivalence curve was established [26]. In a separate study using a tail vein bleeding model and AT‐targeting nanobodies, inhibition of AT activity down to 30% of normal appeared to correspond to an FVIII‐equivalent activity of at least 20% [25]. Based on preclinical data, an FVIII‐equivalence of ≥20% has been proposed to haematologists for fitusiran, despite significant methodological differences between bleeding models and the absence of a standardised calibrator curve [11]. However, such an equivalence does not provide definitive insight into the actual coagulation capacity of patients receiving fitusiran. A key clinical question – particularly in the surgical setting – is whether thrombin generation is normalised. Indeed, decisions regarding the need for additional factor concentrates or bypassing agents rely more on restored haemostatic function than on nominal FVIII‐equivalent values. Thrombotic events observed in patients treated with fitusiran, especially when combined with other pro‐haemostatic agents, further underscore the need for precise and individualised dosing to achieve effective haemostasis without excessive thrombin generation. A detailed analysis of 60 major surgeries conducted in clinical trials revealed that four were successfully managed without any additional factor replacement and without bleeding complications, suggesting that fitusiran may normalise coagulation capacity in certain patients – an unlikely outcome with FVIII levels limited to 20%. Furthermore, most other surgeries required only low doses of factor concentrates (10–20 IU/kg) or bypassing agents (rFVIIa ≤ 45 µg/kg), with excellent or good haemostatic control reported in 97% of cases (Srivastava A, WFH 2025 Comprehensive Care Summit, Dubai, UAE, 21–25 April 2025 – personal communication).

#### 2.5.2 Concizumab

Concizumab FVIII‐equivalent activity was estimated to range between 20% and 50% in a rabbit model of acquired haemophilia using a cuticle bleeding assay [11, 25]. Although FVIII levels in this range are typically sufficient to prevent spontaneous bleeding episodes, clinical trial data have reported treated spontaneous bleeds in some patients receiving concizumab [8]. These findings question the clinical validity and interpretability of FVIII‐equivalence estimations for NFT, particularly given the absence of standardised correlation with thrombin generation or bleeding risk. One possible explanation for this inconsistency lies in the complex distribution of TFPI, which exists in multiple compartments: as a soluble plasma protein, on endothelial surfaces, within platelets and in the extravascular space [27]. Notably, TFPI activity varies by vascular bed, with higher expression on venous endothelium compared to arterial sites [28]. This compartmental and spatial heterogeneity may underlie the site‐dependent efficacy of anti‐TFPI agents such as concizumab, further challenging the concept of a uniform FVIII‐equivalent benchmark for such therapies.

## 3 Discussion

In this article, we critically assess the limitations of the ‘FVIII equivalence’ concept as applied to NFT for haemophilia and evaluate its clinical relevance. Although FVIII‐equivalent estimates can serve as a conceptual tool for comparing the pro‐haemostatic effects of different agents, they may not accurately reflect the true coagulation potential or clinical performance of these therapies. The diverse mechanisms of action, pharmacokinetics and pharmacodynamics of non‐factor agents – such as FVIIIa mimetics, siRNA AT suppression and anti‐TFPI antibodies – challenge the validity of using a single FVIII‐equivalent value across all clinical settings. We argue against the use of FVIII equivalence as a universal surrogate marker of efficacy, and instead propose a shift toward context‐specific laboratory assessment strategies that are aligned with the unique pharmacologic properties and intended clinical use of each agent. This approach may better inform individualised treatment decisions, particularly in high‐risk settings such as surgery or breakthrough bleeding, and support safer and more effective use of novel haemostatic therapies.

In a study by Kizilocak et al. [21], thrombin generation assays were performed in 11 patients with severe haemophilia A receiving emicizumab prophylaxis. All patients exhibited FVIII‐equivalent activity levels greater than 10%, with the majority exceeding 20%. Notably, predicted FVIII levels varied widely and were inversely correlated with body weight, with heavier patients exhibiting lower estimated FVIII equivalence. These findings highlight both the heterogeneity of patient responses to emicizumab and the impact of extrinsic factors such as body weight on coagulation outcomes. However, thrombin generation assays, while valuable, present limitations in replicating in vivo coagulation dynamics. Although they include the complete plasmatic coagulation system, they lack key cellular components – most notably, platelets – as well as the effects of blood flow. In static, platelet‐poor plasma models, FVIIIa is typically the rate‐limiting factor for tenase complex formation. Conversely, in the presence of FVIII mimetics like emicizumab, the limiting component becomes FIXa. Under these conditions, the presence of platelets is essential for the physiological activation of FXI and FIX, which is often absent in the experimental setups used in studies like that of Kizilocak et al. [21]. This limitation may affect the accuracy of FVIII‐equivalence estimations and suggests that thrombin generation results should be interpreted with caution when applied to NFT.

In a recent study, Sefiane et al. [13] assessed the haemostatic efficacy of FVIII, emicizumab and a TFPI‐targeting antibody using four distinct in vivo bleeding models. The study demonstrated considerable variability in the FVIII doses required to reduce blood loss to levels observed in wild‐type mice, ranging from 2.5 IU/kg in the tail vein transection model to 25 IU/kg in the more severe saphenous vein puncture model – a 10‐fold difference. Emicizumab exhibited model‐dependent FVIII‐equivalent activity, approximating 5 IU/kg in the tail clip model and 10 IU/kg in the SVP model. We fully agree with the authors of this study who conclude that their findings support the evidence that a uniform FVIII‐equivalence is unlikely to exist for NFT and cannot be reliably applied across different clinical scenarios.

In conclusion, while in vivo and in vitro models offer valuable insights into the haemostatic efficacy of NFT, they also underscore the limitations of the ‘FVIII‐equivalence’ concept. The biochemical, pharmacokinetic and pharmacodynamic properties of agents such as emicizumab, fitusiran or anti‐TFPI antibodies differ fundamentally from those of FVIII or FIX. Moreover, individual variability in patients’ haemostatic systems further challenges the notion of a universal equivalence. It is increasingly evident that FVIII and FIX possess properties beyond their enzymatic roles in coagulation – such as FVIII potential vascular or inflammatory functions, or FIX extravascular compartmentalisation – that cannot be replicated by mimetic or rebalancing therapies. These unique attributes likely contribute to their clinical effectiveness and are not captured by simple equivalence models. Therefore, equating NFT to specific FVIII or FIX levels risks oversimplifying a complex therapeutic landscape. A more nuanced, context‐specific approach is warranted to guide clinical decision‐making.

## Author Contributions

A.C. wrote the first draft of the introduction. Y.D. wrote the manuscript. M.E.M. critically reviewed and improved the paper. All authors approved the final version.

## Ethics Statement

The authors have nothing to report.

## Conflicts of Interest

Yesim Dargaud has received grants/research support from Bayer, Baxter, Baxalta, Novo Nordisk, CSL Behring, LFB, Pfizer, LeoPharma, Octapharma and Stago; an educational grant from Takeda and honoraria from Bayer, Baxter, Novo Nordisk, CSL Behring, Sobi, Sanofi and Octapharma. Maria Elisa Mancuso acted as consultant, advisor and/or speaker for Shire/Takeda, Bayer, Pfizer, CSL Behring, Novo Nordisk, Grifols, Biomarin, Sobi, Octapharma, Kedrion, Spark Therapeutics, Uniqure, Sanofi, LFB and Roche. Arianna Colombo has no conflict of interest to declare.

## Data Availability

The data that support the findings of this study are available from the corresponding author upon reasonable request.
